# Potential challenges to sustained viral load suppression in the HIV treatment programme in South Africa: a narrative overview

**DOI:** 10.1186/s12981-020-00324-w

**Published:** 2021-01-06

**Authors:** Pascal O. Bessong, Nontokozo D. Matume, Denis M. Tebit

**Affiliations:** 1 AIDS Virus Research Laboratory, HIV/AIDS and Global Health Research Programme, University of Venda, Thohoyandou, 0950 South Africa; 2 Global Biomed Scientific LLC, P.O. Box 2368, Forest, VA 24551 USA

**Keywords:** Universal test and treat, Viral suppression, Treatment outcomes, Potential challenges, South Africa

## Abstract

**Background:**

South Africa, with one of the highest HIV prevalences in the world, introduced the universal test and treat (UTT) programme in September 2016. Barriers to sustained viral suppression may include drug resistance in the pre-treated population, non-adherence, acquired resistance; pharmacokinetics and pharmacodynamics, and concurrent use of alternative treatments.

**Objective:**

The purpose of this review is to highlight potential challenges to achieving sustained viral load suppression in South Africa (SA), a major expectation of the UTT initiative.

**Methodology:**

Through the PRISMA approach, published articles from South Africa on transmitted drug resistance; adherence to ARV; host genetic factors in drug pharmacokinetics and pharmacodynamics, and interactions between ARV and herbal medicine were searched and reviewed.

**Results:**

The level of drug resistance in the pre-treated population in South Africa has increased over the years, although it is heterogeneous across and within Provinces. At least one study has documented a pre-treated population with moderate (> 5%) or high (> 15%) levels of drug resistance in eight of the nine Provinces. The concurrent use of ARV and medicinal herbal preparation is fairly common in SA, and may be impacting negatively on adherence to ARV. Only few studies have investigated the association between the genetically diverse South African population and pharmacokinetics and pharmacodynamics of ARVs.

**Conclusion:**

The increasing levels of drug resistant viruses in the pre-treated population poses a threat to viral load suppression and the sustainability of first line regimens. Drug resistance surveillance systems to track the emergence of resistant viruses, study the burden of prior exposure to ARV and the parallel use of alternative medicines, with the goal of minimizing resistance development and virologic failure are proposed for all the Provinces of South Africa. Optimal management of the different drivers of drug resistance in the pre-treated population, non-adherence, and acquired drug resistance will be beneficial in ensuring sustained viral suppression in at least 90% of those on treatment, a key component of the 90-90-90 strategy.

## Introduction

By the end of 2018, the Joint United Nations Programme on AIDS [1] estimated that about 37.9 million people were infected with HIV worldwide. Although recent UNAIDS data indicate that about 24.7 million people are on antiretroviral therapy (ART), more than double the number reported as recently as 2012 [1], this is still not on track to meet the 30 million by 2020. Of those infected, 7.7 million were in South Africa (SA) [1]. The South African governement introduced treatment for HIV infection in the public health sector in 2004. At that time, only patients with CD4+ T-cell counts of 200 cells μL^−1^ or less were eligible for treatment. Since then, the eligibility criteria to enter the treatment programme has evolved. In light of tangible evidence that treatment significantly reduces HIV transmission at the population level [2, 3]; SA introduced Universal Test and Treat (UTT) in September 2016, a move whereby all tested and prepared persons enter into treatment irrespective of CD4+ T-cell count. The goal of UTT led to the UNAIDS 90-90-90 mantra adopted in 2016, which in practical terms translates to: 90% of the population should know their HIV status, 90% of those with known HIV infection status should be on treatment, and 90% of those on treatment should have sustained suppressed viral loads. The expected outcomes include a significant decrease in mobidity and mortality, and a significant reduction in viral transmission. This is evidenced by the number of AIDS related deaths which has declined by 43% since 2003, the likelihood of viral transmission now reduced to only 4%, and subsequently an end to the epidemic by 2030 [1].

Currently, SA has the largest HIV treatment programme in the world, with about 5 milion people on treatment as of December 2018. In an effort to make ARV available to those in need, major treatment centers are located in large hospitals serving urban dwellers while rural areas have access to primary health care centers within their communities. Treatment initiation occurs mostly at the primary health care level after clinical and psychosocial assessments. At ART initiation, a CD4+ T-cell count is done, then followed-up at six months, and thereafter annually. Meanwhile, a viral load is performed at six months post treatment initiation and then annually [4]. For SA, UNAIDS 2019 data show that 90% of people are aware of their HIV status of which 68% are on treatment and of which 87% are virally suppressed. In essence, 62% of all those living with HIV are on treatment, and 54% of all living with HIV are virally suppressed. Following evidence that dolutegravir, an integrase strand transfer inhibitor, has a higher genetic resistance barrier than non-nucleoside reverse transcriptase inhibitors, in November 2019 the recommended standard initial treatment regimen was switched to the fixed dose combination comprising Tenofovir–Lamivudine–Dolutegravir (TLD), a change from Tenofovir–Lamivudine–Efavirenz [5, 6].

There are several key assumptions in the operationalization of UTT. These assumptions include: firstly, that all persons diagnosed and accessing treatment have viruses which are susceptible to the recommended first line treatment regimen. In this assumption, the scenario is that the prevalence of circulating drug resistant viruses in the pre-treated population is negligible to cause a significant negative impact on treatment outcomes. Secondly, that the proportion of patients with prior exposure to treatment from one locality and re-initiating treatment in another locality, without disclosing their prior exposure, is negligible to impact on treatment outcomes. Thirdly, there is a high level of adherence to treatment coupled to minimal acquired resistance to achieve sustained viral load suppression. Fourthly, the pharmacokinetics and pharmacodynamics of antiretrovirals is optimal in the population; and lastly, that in a country, such as South Africa, where there is appreciable concomitant use of ARV and herbal preparations, the efficacy of ARV is not compromised.

As a result of these assumptions, patients are not assessed for the following prior to treatment initiation: exposure to ARV using objective indicators such as ARV in plasma or hair, harbouring of resistant viruses, optimal pharmacokinetics and pharmacodynamics parameters, and concomitant use of traditional, complementary and alternative medicines (TCAM) with a view to achieve optimal outcomes from ART. The SA national treatment guidelines do not recommend these for patients entering treatment programmes. So, it is important to begin to understand how these assumptions may impact UTT in SA.

In the absence of a cure or preventive vaccine for HIV, the judicious use of available treatment options in any treatment programme is important and desirable to maximize benefit for patients both at the individual and at the population level, thereby contributing towards a reduction in viral transmission. This narrative aims to examine potential challenges to the expected outcomes of the UTT programme in SA. Specifically, the questions that this review intends to explore are: (1) is there evidence for an appreciable level of drug resistant viruses in HIV infected patients who are not yet on treatment? (2) Are there protocols to identify prior exposure to antiretrovirals for those entering a treatment programme? (3) Is there evidence of archived drug resistant provirus in those who are not yet on treatment? (4) Is there evidence, from more objective markers such as drug concentrations in plasma or hair, to detect the levels of adherence that support a sustained viral load suppression? (5) How does herb–drug interaction impact on the efficacy of ART in SA? Answers to these questions are important in identifying potential challenges, designing interventions, and improving outcomes of UTT in SA.

## Methods

To narrate whether or not HIV infected persons entering treatment programmes in South Africa do harbour resistant viruses, to such an extent as to reduce the efficacy of the treatment regimen, at least at the population level; and whether pre-treated persons harbour resistant viruses (resistance provirus), published literature in the English language which examined the presence of drug resistant viruses in HIV infected persons who are not on treatment were examined. Literature was sourced in PubMed and Web of Science databases, with no limitation by date of publication. Search terms and criteria implemented in PubMed were: ((HIV[Title/Abstract]) AND "South Africa"[Title/Abstract]) AND resistance [Title/Abstract]; with HIV used as a MeSH Terms.

For the Web of Science, we applied the ‘Advance search’ option for all databases using the following search terms TOPIC: (HIV) OR (HIV-1) OR (Human immunodeficiency virus) OR (Human immunodeficiency virus type 1) AND Topic: (Resistance) AND Topic: (“South Africa”). Additional searches were done to specifically identify data on drug resistant viruses in the drug in-experienced population from each of the nine provinces of South Africa. The outputs of the search of both PubMed and Web of Science were imported into Mendeley and an internal search using the following keys words to generate an output specific for drug resistance studies in the drug inexperienced population: naïve, pre-treatment, in-experienced, and untreated.

Another internal search was done to identify papers on adherence using more objective methods such as drug concentrations in hair and plasma, as well as to identify studies that used proviral DNA to detect HIV drug resistance in the pre-treated population. The search terms used included: resistance, adherence, hair, plasma, and DNA.

Drug pharmacokinetics and pharmacodynamics impact the efficacy of ARV. To estimate the degree of efficiency of ARV pharmacokinetics and pharmacodynamics in the SA population, published data on these topics were reviewed. The search terms and criteria implemented in PubMed were: (((("Human immunodeficiency virus") OR (HIV[Title/Abstract])) OR (HIV-1[Title/Abstract])) AND (((((((MDR1[Title/Abstract]) OR (CYP2B[Title/Abstract])) OR ("Cytochrome p450"[Title/Abstract])) OR ("drug metabolism"[Title/Abstract])) OR ("drug transport"[Title/Abstract])) OR (Pharmacodynamics[Title/Abstract])) OR (pharmacokinetics[Title/Abstract]))) AND ("South Africa"). The MeSH terms included: transportation; metabolic networks and pathways; membrane transport proteins; biological transport; HIV; metabolism; Subheading: metabolism. The terms and criteria for Web of Science, Advance search option included: Web of Science, Advance search option included: TOPIC: (HIV) OR (HIV-1) OR (Human immunodeficiency virus) OR (Human immunodeficiency virus type 1) AND TOPIC: (Drug metabolism and transport) OR (“Cytochrome p450”) OR (CYP2B) OR (MDR1) OR Pharmacodynamics OR Pharmacokinetics AND Topic: (“South Africa”).

**Fig. 1:**
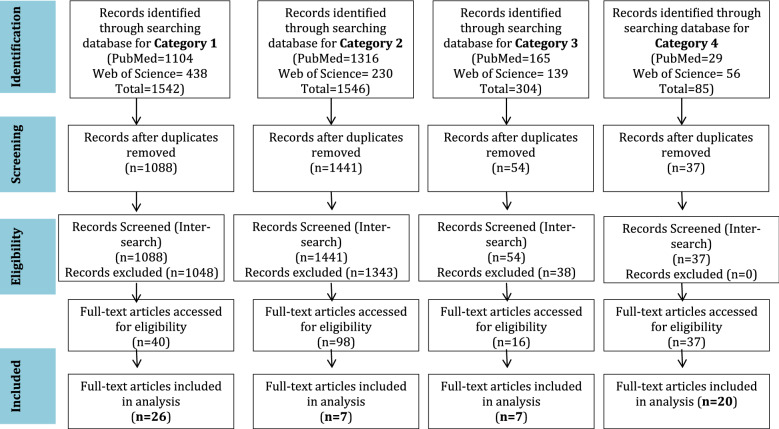
Prisma flow diagram on the identification and screening of articles for eligibility and inclusion for all search categories. Category 1: searched articles on drug resistant viruses in the pre-treated population in South Africa. Category 2: searched articles on ART adherence using ARV in hair and plasma as a marker. Category 3: searched articles on ARV Pharmacodynamics and Pharmacokinetics. Category 4: search articles on alternative medicinal use by individuals on ARV

The concurrent use of alternative medicine in the form of herbal preparations with ARV may lead to suboptimal adherence and drug interactions with the potential to affect treatment outcome. To estimate the concurrent use of medicinal herbs in South Africa, published data on these topics were also reviewed. The search terms used in both PubMed and Web of Science were; (((("alternative medicine") OR "medicinal plants") OR herb*) AND "Antiretroviral therapy") AND "South Africa" and TOPIC: (alternative medicine) OR (medicinal plants) OR (herb*) AND (Antiretroviral therapy) AND (South Africa) for both PubMed and Web of Science respectively. Figure 1 represents the PRISMA strategies employed in extracting and selecting papers for analysis of resistant viruses in the pre-treated population, pharmacokinetics and pharmacodynamics of ARV, adherence, and use of complementary medicine by individuals on ARV in South Africa.

## Results and discussion

### Levels of drug resistant viruses in the pre-treated population

A total of 26 articles met the inclusion criteria and were analyzed for levels of drug resistant viruses in pre-treated patients in South Africa (Fig. 1). The World Health Organization categorizes the levels of HIV drug resistance in a pre-treated population as low (< 5%), moderate (5–15%) and high (> 15%). Our observation shows that there is clear heterogeneity in the prevalence of resistant viruses in the pre-treated population across and within provinces in South Africa. Between 2001 and 2016, 22 studies investigated clinically relevant drug resistance mutations in the pre-treated population at the provincial (n = 18) and national levels (n = 4) (Table 1). The national surveys show a DR mutation prevalence of 0.9–9% falling within the moderate range (Table 1). In almost all the studies, the order of DRM was NNRTI > NRTI > PI with respective prevalence of moderate, low and low (Table 1). Individual studies carried out within the provinces with pregnant mothers (PMTCT) or commercial sex workers as participants show higher levels (> 15%) of DRMs, while all other studies fall within the 5–10% range (Table 1). For example, in a drug resistance threshold survey with samples collected in 2002 and 2004, a prevalence of less than 5% was reported in Gauteng Province in each of the respective years [7]. These mutations included NNRTI (K103N) and NRTI (T69D, K70R). Similarly, analyses conducted at 9 survey sites, five sites in KwaZulu-Natal province and four in Gauteng Province between 2005 and 2009 indicated a low (< 5%) but stable prevalence in Gauteng Province, and a potentially increasing prevalence (5–15%) of NNRTI (K103N, V106M, K101P) mutations in KwaZulu-Natal Province [8]. Protease inhibitor resistance mutations (M46I and I85V) were also low (< 5%) for samples collected in 2007 and 2009 from Gauteng and KwaZulu-Natal respectively [8]. Samples were collected from individuals who met the inclusion criteria for the WHO guidelines for the classification of transmitted drug resistance, and recent infections were selected based on the BED EIA HIV-1 incidence test. Transmitted drug resistance, using Sanger sequences, was significant for Gauteng Province in 2008 (p = 0.009) and in 2009 (p = 0.040), and only for 2009 in Kwazulu-Natal Province (p = 0.029). More recently, Steegen et al. [9] conducted a prospective cross-sectional survey, employing probability proportional to size sampling for drug resistance from each of the nine provinces of SA, between March 2013 and October 2014. Overall, using Sanger generated sequences and analysed by the Stanford Calibrated Population Resistance tool, surveillance drug resistance mutations (SDRM) were detected in 9% (95% CI 6.1–13.0%) of the study population, with a higher prevalence of NNRTI (K103N > Y181C > V106M) followed by NRTI (K65R, M184V) and only 2 PI (V32I and L90M) mutations. Of note is the fact that thymidine analogue mutations (TAMs) were not found in this study population probably due to the switch in 2010 from d4T and AZT to TDF as the backbone of first line therapy. Recently, in estimating the prevalence of provincial and national drug resistance, Hunt and colleagues [10] reported moderate levels (5–15%) of NNRTI transmitted drug resistance in samples collected in 2010 and 2012 from Eastern Cape, Free State and KwaZulu-Natal Provinces. All nine provinces considered, the prevalence of NNRTI was 5.4% (95% CI 3.7–7.8%), with K103N and V106M being the most frequently detected mutations. Estimates for NRTI were 1.1% (95% CI 0.5–2.4%) and 0.6% (95% CI 0.1–1.6%) for PI. Other reports have suggested increasing levels of drug resistance mutations in the pre-treated population in SA. In an analysis of pooled *pol* sequences between 2000 and 2015, Chimukangara et al. [11] found an increase of reverse transcriptase inhibitor drug resistance mutations from less than 5% prior to 2009 to about 12% in 2015, with at least one-fold increase in mutations conferring resistance to NNRTI and NRTI.

**Table1:** Summary of provincial and national HIV-1 surveillance drug resistance mutation (SDRM) studies in South Africa

Population type	Sampling year	Province	Number of participants	DR prevalence (%)	NNRTI (%)	NRTI (%)	PI (%)	References
Pre-treated individuals	2013–2014	Eastern Cape	25	16	16	4	0	[9]
	2013–2014	Free State	25	12	12	4	0	[9]
	2006	Free State	390	2.3	3.3	0.8	0.5	[19]
	2016	Gauteng	95	27.4	24.2	3.1	0	[22]
	2002–2004	Gauteng	113	4.2	2	2	0	[7]
	2012–2016	KwaZulu-Natal	1148	12.8	8.8	2	1.4	[15]
	2013–2015	KwaZulu-Natal	1845	11.5	10	3.2	1.2	[18]
	2010–2012	KwaZulu-Natal	701	5.1	4.6	1.4	0	[17]
	2010–2011	KwaZulu-Natal	326	8	7.1	2.1	0.6	[16]
	2009	KwaZulu-Natal	44	2	2	0	0	[14]
	2008	Limpopo	80	2.5	1.2	1.2	0	[12]
	2008	Limpopo	54	9.3	0	7.4	1.8	[12]
	2009	Mpumalanga	51	5.9	5.9	0	0	[9]
	2013–2014	North West	13	7.7	7.7	0	0	[9]
	2013–2014	Northern Cape	1	1	0	0	0	[9]
	2008–2010	Western Cape	58	5.2	3.4	1.7	1.7	[9]
	2002–2007	Western Cape	120	4.2	1.6	3.3	0	[20]
	2002–2004	Western Cape	140	3.6	2.1	2	0	[21]
PMTCT-exposed	2011	Gauteng	155	56.8	56.8	14.8	1.3	[24]
		Gauteng	75	24	24	10.7	1.3	
	2005–2007	Gauteng	255	31	27	7.5	1.6	[25]
	2005–2006	Gauteng	226	30	25.7	3.1	1.3	[84]
	2007–2009	Western Cape	47	6.4	6.4	0	0	[26]
Data from National surveys in South Africa								
Pre-treated adults	2013–2014	All provinces	277	9	8.3	2.5	0.7	[9]
Pre-treated adults	2010–2012	All provinces	770	7	5.4	1.1	0.5	[10]
Pre-treated adults	2005–2009	KwaZulu Natal and Gauteng	354	3.1	2.3	1.1	0.6	[8]
PMTCT-Exposed	2010–2013	All provinces	220	51	51	5	0.9	[30]

**Fig. 2:**
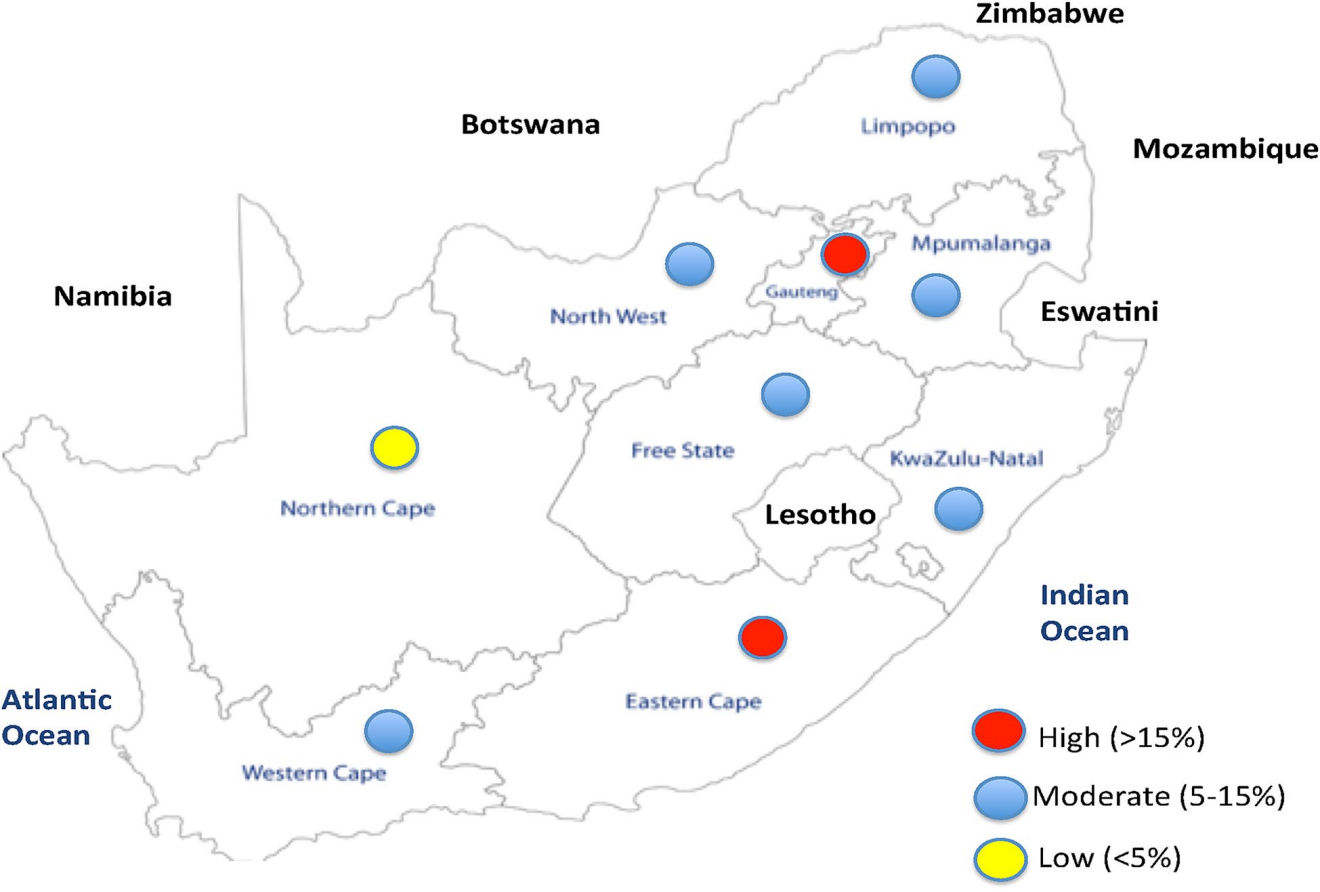
Map of South Africa showing the highest level of drug resistance in at least one pre-treated population in the provinces. The position of the circles is not indicative of a specific geographical location

Provincial-based studies have also underlined the heterogeneity in the prevalence of resistance viruses across the national landscape (Fig. 2). Using Sanger sequencing to analyse samples from individuals presenting for voluntary counselling and testing in Limpopo Province in 2008, a less than 5 and 9% of TDR in the Waterberg and Capricorn districts respectively was reported [12]. An examination of samples collected from rural sites between 2000 and 2010 in KwaZulu-Natal found TDR of 6% in 2002 with a decline to < 5% thereafter [13]. Elsewhere in KwaZulu-Natal, resistance threshold studies with samples collected in 2005 and 2009 reported a similar outcome of less than 5% prevalence [14]. However, a recent NGS analysis with a 5% sequence depth threshold documented about 13% viral genetic resistance in a rural population in KwaZulu-Natal Province [15]. This rise in TDR prevalence among drug naïve subjects could be explained partially by the sensitivity of the NGS compared to Sanger sequencing whose lower limit of sensitivity is approximately 20%. Earlier, in a cross-sectional study of reproductive-aged women from 7 clinical sites in Durban (KwaZulu-Natal), Sanger sequencing revealed that 7.4% had NRTI, NNRTI or PI drug resistance mutations prior to treatment initiation [16]. There are indications that the level of resistant viruses is increasing in KwaZulu-Natal Province in a study by Manasa et al. [17] which found no DRM in 2010, 4.7% in 2011 and 7.1% in 2012. Also in KwaZulu-Natal, a prevalence of 9.2% of surveillance drug resistance mutations was noted in individuals less than 15 years of age sampled between 2013 and 2014; and 11% in individuals between 15 and 49 years of age sampled between 2014 and 2015, with no prior exposure to antiretroviral therapy [18].

In the Free State Province, a prevalence of 2.3% has been reported in adults being prepared for treatment initiation [19]. Among treatment-naive patients, prevalence of primary resistance was 2.5% in Cape Town (Western Cape Province) from sampling done in public sector healthcare facilities between 2002 and 2007 [20]. Similarly, a 3.6% genetic drug resistance was observed in a cross-sectional population from samples collected prior to the commencement of the national treatment programme in Cape Town [21]. On the other hand, drug resistance prevalence of about 27% has been reported in a cross section of drug naïve female sex workers in Soweto, Gauteng province [22]. Provinces in which at least one study has reported a moderate or high level of HIV drug resistance in a pre-treated population are shown in Fig. 2.

Prevention of mother-to-child-transmission (PMTCT)  has been highly successful in SA with only about 3% of children born to HIV infected mothers contracting the infection [23]. Although PMTCT programmes have led to a significant decrease in transmission, the rate of DR among newly infected children remain high (> 15%). For example, a report from a 2011 study performed with samples collected between 2005–2007 among newly infected 2 year olds in Johannesburg who had been exposed to maternal or infant ART, suggests that of those who get infected, as high as 57% harbour viruses resistant to NNRTI, 15% to NRTI and 1.5% to PIs [24]. The prevalence of DRM among PMTCT unexposed children was much lower; NNRTI (24%), NRTI (11%) and PI (1.3%). In a similar study among sdNVP exposed children less than 2 years old who were about to be initiated on ART in Gauteng Province, NNRTI DRM (mostly Y181C) were found to decrease with the child’s age. Notable, up to 68% of 6–12 month olds carried NNRTI mutations while only 16% of NNRTI resistant mutations was detected in children aged 18–24 months old [25]. A study by van Zyl et al. [26] reported a high (16.3%) NNRTI but no NRTI or PI SDRM in children < 18 months old in the Western Cape Province; while another study indicated a moderate prevalence of 11% in children infected vertically [27]. The effectiveness of sdNVP may be compromised by pre-exposure to the same drug. A study by Martinson et al. [28] found that at 6 weeks postpartum, 11.1% of children whose mothers had previously been exposed to sdNVP were HIV-1 positive compared to 4.3% for those without sdNVP exposure. In terms of DRM, 25% of mothers previously exposed to sdNVP carried either K103N or Y181C mutations compared to 12.5 non-exposed mothers who had only Y181C mutations at baseline. In general, PIs have very low DRM among children and have been suggested as a drug of choice for this young population. A study that exclusively examined 2,000 PI naïve sequences collected over a 14 year period detected less than 1% of PI resistance mutations [29]. With the current guideline replacing efavirenz in the standard first line regimen with dolutegravir, it would be of interest to see how this impacts the prevalence of resistance mutations in the pre-treated population. Since most of the studies used the Sanger approach for sequencing, it will be interesting to see if applying the more sensitive next-generation sequencing approach will present a different picture.

Several drivers may be contributing to drug resistance in the pre-treated population. Prevention of mother-to-child transmission programmes have been highly successful; however, this has led to a certain extent of resistance in infants [30]. There is increasing exposure to antiretrovirals through pre-exposure and post-exposure prophylaxes in many African communities [31]. Antiretrovirals are also used in managing pathologies such as HBV [32, 33]; and their use as narcotics has been reported [34]. The detection of drug resistant viruses in non-treated persons is generally assumed to be due to an infection by a resistant virus from an individual on treatment. However, the level of resistance due to de novo changes is not well understood.

Overall, current data show that moderate levels of drug resistance has been reached in certain communities and populations in SA. Sentinel surveillance targeting these communities and populations are required for interventions relevant to local realities.

### Prior exposure to ARV and adherence to treatment

It is imperative that the ARV prior exposure history of individuals entering a treatment programme is understood accurately for better management. Generally, there is evidence of self-reporting on prior exposure to ARV, particularly for women of child-bearing age if they have been involved in a PMTCT programme. But there is apparently no evidence of approaches or systems aimed at determining whether any other individual entering an ART programme has been on treatment elsewhere in the absence of a self-report. In particular, males entering a treatment programme are presumed naïve by clinical staff. This may be of concern since, using validated methods, significant amount of drug concentrations has been detected in the hair or plasma of individuals, including males, who self-reported that they have no prior exposure to antiretrovirals upon treatment initiation [35]. The question that arises is whether individuals in whom drug concentrations are detected in hair or plasma are knowingly approaching a different treatment programme to re-initiate. We identified eight studies that reported on the use of hair or plasma as more objective and accurate methods to estimate adherence and sustained drug concentrations in patients (Table 2); [36–41]. These approaches could also be employed to detect prior exposure to antiretrovirals. A drawback though is that these methods are expensive, in their requirements for equipment and technical personnel. Therefore, point-of-care devices on these approaches are highly desirable [42].

**Table 2:** Relevant studies describing the use of hair and plasma as methods to estimate adherence and sustained drug concentrations in patients in South Africa

Title of study	Province of study	Main study outcome	References
Correlation of hair and plasma efavirenz concentrations in HIV-positive South Africans	Western Cape	Median efavirenz concentrations for extensive, intermediate and slow metabolising genotypes were 3.54, 5.11 and 10.66 ng mg^−1^, respectively. A strong correlation was observed between the efavirenz concentrations measured in hair and plasma samples (Spearman’s correlation coefficients, 0.672–0.741, *p* < 0.0001). This shows hair could be used a matrix for measuring antiretroviral concentrations as markers of adherence	[41]
A comparison of plasma efavirenz and tenofovir, dried blood spot tenofovir-diphosphate, and self-reported adherence to predict virologic suppression among South African women	Western Cape	Dried blood spot TFV-DP [0.926 (95% CI: 0.876 to 0.976)] had a higher area under the curve than plasma TFV [0.864 (0.797 to 0.932); p = 0.006], plasma EFV [0.903 (0.839–0.967)] was not significantly different from DBS TFV-DP (p = 0.138) or plasma TFV (p = 0.140); all ARV assays performed better than self-report. The association of EFV, TFV, and DBS TFV-DP were seen as strong predictors of virologic suppression. Data shows that EFV or TFV assays have potential for development as point-of-care assays for use in more objective adherence measurement in resource-limited settings	[42]
A validated liquid chromatography tandem mass spectrometry method for the analysis of efavirenz in 0.2 mg hair samples from HIV-infected patients	Western Cape	Using ten times less hair than in a previously published method, the lower limit of quantitation (LLOQ) was validated at 0.625 ng mg^−1^. This paper perhaps reports the first quantitative method for the determination of efavirenz in hair in South Africa. The validated method was used to successfully monitor efavirenz concentration in hair collected from HIV-infected patients	[85]
CYP2B6*6 and CYP2B6*18 predict long-term efavirenz exposure measured in hair samples in HIV-positive South African women	Western Cape	The predictive value of EFV levels in hair and selected variants in *CYP2B6* on virologic treatment outcomes was assessed. Previously described alleles (CYP2B6*2, CYP2B6*5, CYP2B6*6, CYP2B6*17, and CYP2B6*18), as well as two novel alleles (CYP2B6*31 and CYP2B6*32), were detected in the study. Compared to noncarriers, individuals homozygous for CYP2B6*6 had ∼109% increased EFV levels in hair (*p* = 0.016) and CYP2B6*18 heterozygotes demonstrated 82% higher EFV hair levels (*p* = 0.0006)	[39]
Random lopinavir concentrations predict resistance to lopinavir-based antiretroviral therapy	Western Cape and KwaZulu Natal	A random lopinavir concentration above the recommended minimum trough of 1 μg mL^−1^ [adjusted odds ratio (aOR) = 5.81, 95%, CI: 2.04–16.50; p = 0.001] and male sex (aOR = 3.19, 95% CI 1.22–8.33; p = 0.018) were predictive of the presence of at least one major PI resistance mutation. Random lopinavir concentrations of < 1 μg mL^−1^ had a negative predictive value of 91% for major PI resistance mutations. Random lopinavir concentrations are strongly associated with the presence of major PI resistance mutations	[38]
Plasma lopinavir concentrations predict virological failure in a cohort of South African children initiating a protease-inhibitor-based regimen	Gauteng	Median (IQR) lopinavir concentrations were 8.00 mg L^−1^ (4.11–12.42) at median (IQR) 3.50 h (2.67–4.25) after the dose. The hazard of viral load > 400 copies mL^−1^ was increased with lopinavir concentrations < 1 versus ≥ 1 mg L^−1^ (adjusted hazard ratio 2.3 [95% CI 1.63, 3.26]), and lower height-for-age z-scores. Low lopinavir concentrations (< 1 mg L^−1^) were associated with viraemia in children. This measure could be used as a proxy for adherence and to identify children more likely to fail treatment	[37]
Low lopinavir plasma or hair concentrations explain second line protease inhibitor failures in a resource-limited setting	Western Cape	The probability of failure with lopinavir plasma concentration > 1 μg mL^−1^ or hair concentrations > 3.63 ng mg^−1^ for virologic failure were 86 and 89%; and positive predictive values of low concentrations 73 and 79%, respectively, whereas all virologic failures with HIV RNA loads above 1000 copies mL^−1^, of patients without protease inhibitor resistance, could be explained by either having a low lopinavir concentration in plasma or hair	[36]

More objective mechanisms to identify, for example, a patient who approaches the public health sector from the private sector or vice versa, without disclosing prior treatment are necessary. One such method could be the establishment of a centralized national treatment database accessible to authorized healthcare practitioners to support the rationalization of the starting treatment regimen.

Once on treatment, adherence to treatment regimen is fundamental to reduce the chance and speed of drug resistance development, and to achieve outcomes such as undetectable viral load, significant increases in CD4+ T-cells, and reduced morbidity. A study by Myer et al. [43], showed that about 90% of cases with increased viral loads (> 1000 copies µL^−1^) in a cohort of pregnant women in Cape Town was due to non-adherence to ART as opposed to the presence of pre-treatment drug resistance mutations. Earlier, Hunt et al. [44] in a study in KwaZulu-Natal, identified male gender and individuals entering treatment at an advanced stage of disease as groups for which intensified adherence monitoring may be needed. Perhaps less than optimal adherence is accounting for the currently observed modest level of viral suppression (54%) in the treated population [1]. While data from Hoffmann et al. [45] supports intensified adherence for optimal benefit from first-line use and to minimize the risk for the development of cross-resistance, other studies have advocated for improved adherence to enable patients switching to second line regimens to continue to benefit from treatment [36, 46].

For patients re-initiating treatment, the national treatment guidelines recommend the taking of a thorough history on the previous regimen and duration, noting the reasons for stopping treatment, side effects, and a review of previous viral load data, to inform next steps. However, the guidelines do not emphasize drug resistance testing and mental health assessment. Resistance testing has been shown to be of significant value in managing patients re-initiating treatment even after three months of treatment interruption [47], and more so if deep sequencing is used [48]; and mental health intervention could be critical for improved adherence [49].

Several determinants of non-adherence to ART in the general population or designated populations in South Africa have been proposed. These include psychosocial factors such as stigma, alcohol abuse, and mental health; socio-economic factors such lack of information, unemployment, long distance from clinics; drug toxicities, and deficiencies in the health system such as drug stock-outs and inadequate patient management [50–54]. The significant adversity from non-adherence is highlighted in a model analysis which predicts a spike in HIV infections in South Africa if a decrease in adherence is coupled with an increase in the rate of loss to follow-up in the test and treat programme [55]. There is a myriad of challenges to adherence and an approach whereby the risk of non-adherence for each patient is regularly assessed for the best intervention is the most plausible for an optimal outcome [56].

### Host genetics and ARV pharmacokinetics and pharmacodynamics

The variability in host genetics impacts pharmacokinetics and pharmacodynamics of ARV. This, in turn may lead to differences in plasma drug concentration, which can impact drug resistance development or adverse effects [57, 58]. Our search revealed only seven (n = 7) articles relevant to the relationship between host genetics of the ethnically diverse South African population and pharmacokinetics and pharmacodynamics of ARV (Table 3). In a study to determine the frequency of genetic variants associated with the pharmacokinetics of ARV in South Africans, there were differences in 53 variants in allele and genotype frequencies between designated South African coloureds and South African Blacks; with 24.5% of these being of clinical relevance impacting on the pharmacokinetics of tenofovir and efavirenz [59]. Similarly, a study by Swart and colleagues [60] reported differences in allele frequencies among South Africans with African, Caucasian and Asian populations. Specifically, they reported that CYP2B6 516 T and 785G (*6) and CYP2B6 983C (*18) alleles are significantly associated with high plasma efavirenz levels, and G-G-A-T-C and A-G-A-T-C haplotypes showed significantly lower levels of efavirenz. The authors suggest screening for CYP2B6 516G > T SNP, which has a high specificity and positive predictive value, as a marker for efavirenz dosage for individuals homozygous for the variant. In another study, polymorphisms in the CYP2B6 gene associated with extensive, intermediate and slow metabolizers of efavirenz were identified in black adults and children [61]. Studies by Reay and colleagues also suggest that CYP2B6 haplotypes could be used to predict EFV plasma concentrations in black South African children [62, 63]. These findings though should be seen in the backdrop of recent changes (November 2019) in which, dolutegravir replaced efavirenz because of the former’s high genetic resistance barrier. It is expected that data on the relationship of dolutegravir and host genetics will soon begin to emerge. Examination of the MDRI gene among black South Africans did not find significant difference between immune recovery and decline in viral load with MDRI genotypes [64]. Similarly, a univariate two-way analysis of variance did not show any effect of ethnicity on CD4+ T-cell count in response to ART, but T129C and G2677A polymorphisms in the ABCB1 gene showed a marginal effect on immune recovery [65].

**Table 3:** Relevant studies on the relationship between host genetics and ARV transport and metabolism in the ethnically diverse South African population

Title of study	Province of study	Main study outcomes	References
Pharmacogenetics of antiretroviral drug response and pharmacokinetic variations in indigenous South African populations	Western Cape and Gauteng	Fifty-three variants had significant differences in allele and genotype frequencies when comparing South African colored and black African groups. Thirteen of these had strong clinical annotations, affecting efavirenz and tenofovir pharmacokinetics	[59]
CYP2B6 haplotype predicts efavirenz plasma concentration in black South African HIV-1-infected children: a longitudinal pediatric pharmacogenomic study	Gauteng	The CYP2B6 c.516T/T genotype showed significantly higher EFV plasma concentrations (p < 0.001) compared to non 516T-allele carriers. The minor allele frequencies (MAF) for CYP2B6 c.516T, c.785G, c.983C, and c.1459T were 0.410, 0.408, 0.110, and 0.000 respectively. The haplotype T-G-T presented with significantly increased EFV plasma concentrations compared to the reference G-A-T haplotype at 1, 3, and 24 months (p = 0.009; p = 0.003; p = 0.001). This suggests, the T-G-T haplotype predisposes a risk of EFV plasma concentrations > 4 lg mL^−1^	[63]
Pharmacogenetics of plasma efavirenz exposure in HIV-infected adults and children in South Africa	Not indicated	Median (IQR) mid-dose efavirenz concentrations observed were 1.44 (1.21–1.93) μg ml^–1^, 2.08 (1.68–2.94) μg ml^–1^ and 7.26 (4.82–8.34) μg ml^–1^ for extensive, inter- mediate and slow metabolizers, respectively. In univariate analyses, a model that included composite genotype best predicted efavirenz concentrations (β = 0.28, 95% CI 0.21, 0.35, p = 2.4 × 10^–11^). Among individual CYP2B6 polymorphisms, 516G → T best predicted efavirenz concentrations (β = 0.22, 95% CI 0.13, 0.30, p = 1.27 × 10^_6^). There was also an association with 983T → C (β = 0.27, 95% CI 0.10, 0.44, p = 0.002) and 15582C → T (β = 0.11, 95% CI 0.01, 0.22, p = 0.04). Associations were consistent in adults and children	[61]
High predictive value of CYP2B6 SNPs for steady-state plasma efavirenz levels in South African HIV/AIDS patients	Gauteng	CYP2B6 516T and 785G (*6) and CYP2B6 983C (*18) alleles were observed to be significantly associated with high plasma efavirenz levels. CYP2B6 A–G–A–C–C and A–T–G–T–C haplotypes (with respect to CYP2B6 136A>G; CYP2B6 516G>T; CYP2B6 785A>G; CYP2B6 983T>C; and CYP2B6 1459C>T) were associated with higher levels of efavirenz, whereas G–G–A–T–C and A–G–A–T–C haplotypes showed significantly lower efavirenz levels	[60]
Prevalence of MDR1 C3435T and CYP2B6 G516T polymorphisms among HIV-1 infected South African patients	Limpopo	Analysis of population-based sequences of MDR1 revealed a frequency of 89 and 11% of C and T alleles respectively (n = 197; X^2^ = 0.974; p = 0.324). Restriction fragment length polymorphism analysis of the CYP2B6 gene revealed a prevalence of 9.5% of GG, 78.4% of GT and 12.1% of TT genotype (n = 199; X^2^ = 65.204; p = 0.00). No significant difference between immune recovery and decline in viral load (n = 53) was observed with genotype after repeated calculations of analysis of variance	[64]
Influence of CYP2B6 516G>T polymorphism and inter-occasion variability (IOV) on the population pharmacokinetics of efavirenz in HIV-infected South African children	Gauteng	EFV concentrations below 1 μg mL^−1^ accounted for 18% (116/649), concentrations > 4 μg mL^−1^ accounted for 29.5% (192/649) and concentrations within the therapeutic range (1–4 μg mL^−1^) represented 52.5% (341/649) of all the samples determined. Age, weight and CYP2B6 G516T genotype were included in a model with population estimates for apparent clearance determined as 2.46, 4.60 and 7.33 L h^−1^ for the T/T, G/T and G/G genotype groups respectively	[62]
A pharmacogenetic study of CD4+ T-cell recovery in response to HIV antiretroviral therapy in two South African population groups	Western Cape	Considering CD4+ T-cell count, univariate two-way analysis of variance showed no apparent effect of ethnicity on immune recovery in response to ART. Univariate one-way ANOVA testing revealed a noticeable effect of genotype on immune recovery for T-129C (p = 0.03) and G2677A (p < 0.01) polymorphisms in the ABCB1 gene	[65]

Overall, there is limited data to guide our understanding, to an appreciable extent, on the relationship between host genetics of the ethnically diverse South African population and pharmacokinetics and pharmacodynamics of ARV, and how this impacts viral suppression in the era of test and treat.

### Traditional, complementary and alternative medicine, adherence to ARV, and treatment outcome

The use of traditional, complementary and alternative medicine (TCAM) in the treatment of HIV infection is quite common in low- and middle-income countries, including South Africa, and herbal preparations are taken concurrently with ARV [66–69]. Since optimal adherence to ART is key to sustained viral suppression, the concern has been how the concomitant use of TCAM impacts adherence to ART. We extracted 20 studies relevant to the association between the use of TCAM and ART outcomes in South Africa (Table 4). Earlier, a multivariate regression analysis involving participants from public sector hospitals in KwaZulu-Natal showed that although TCAM usage reduced in the study population upon initiation into ART, adherence to ART was significantly impacted among those who continued using TCAM [70].This observation was further emphasized in a prospective study [71]; in which the use of TCAM was observed to be significantly associated with virologic failure [72]. Understanding the use of TCAM during counselling sessions on adherence to ART can be missed since patients may withhold this information from healthcare workers for fear of lack of understanding and disclosure of their HIV positive status to close relatives and friends [73, 74]. A study by Loeliger and others [75] proposed a strengthening of collaboration between community health care providers and traditional healthcare providers as a means of sustaining adherence to ART; and a recent study has shown that adherence to ART could be improved even among HIV infected traditional healthcare practitioners [76].

**Table 4:** Studies reviewed on the association between the use of alternative medicines and ART outcomes in South Africa

Title of study	Province of study	Main study outcomes	References
Improved adherence to anti-retroviral therapy among traditionalists: reflections from rural South Africa	Limpopo	A strategy was developed to contribute to improved adherence and a reduction of internalized stigma among HIV infected traditionalists in Waterberg district, South Africa	[76]
The in-vitro and in-vivo effects of *Hypoxis hemerocallidea* on indinavir pharmacokinetics: modulation of efflux	Not indicated	The combined effect of efflux and metabolism inhibition by *H. hemerocallidea* has clinical significance on indinavir pharmacokinetics	[78]
Traditional, complementary and alternative medicine use in Sub-Saharan Africa: a systematic review	Sub-Saharan Africa	Traditional, complementary and alternative medicine use in sub-Saharan Africa is significant	[66]
Herbal slimming formulations or remedies interact with antiretroviral therapy	Not indicated	There is potential for reduced or increased serum antiretroviral drug concentrations. Subtherapeutic drug levels could lead to unsatisfactory viral suppression. Herbal products may also contain compounds that interfere with the absorption of antiretrovirals	[86]
Concurrent use of antiretroviral and African traditional medicines amongst people living with HIV/AIDS (PLWA) in the eThekwini metropolitan area of KwaZulu Natal	KwaZulu-Natal	Concurrent ARV and ATM use is quite low when compared to ATM use before HIV diagnosis and after HIV diagnosis but before initiation with ARV	[87]
Traditional, complementary and alternative medicine use by HIV patients a decade after public sector antiretroviral therapy roll out in South Africa: a cross sectional study	KwaZulu-Natal	The use of traditional, complementary and alternative medicine is prevalent amongst a small proportion of HIV infected patients attending public healthcare sector antiretroviral clinics	[67]
Antiretroviral therapy initiation and adherence in rural South Africa: community health workers' perspectives on barriers and facilitators	KwaZulu-Natal	There are a number of factors associated with non-adherence, which includes a dilemma between ART and alternative medicine use	[75]
Inhibition of CYP2B6 by medicinal plant extracts: implication for use of efavirenz and nevirapine-based highly active anti-retroviral therapy (HAART) in resource-limited settings	Not indicated	There is a high probability that standard doses affect drug plasma concentrations, which could lead to toxicity, when drugs that are metabolized by CYP2B6, are co-administered with herbal medicines	[88]
Concurrent use of traditional medicine and ART: perspectives of patients, providers and traditional healers in Durban, South Africa	KwaZulu-Natal	Some patients do not view TAM as an alternative to ART; rather, employ TAM and ART for distinctly different needs	[89]
The potential of *Sutherlandia frutescens* for herb–drug interaction	Not indicated	Herb–drug interactions have an effect on enzymes responsible for ART drug metabolism and transport	[90]
Why HIV positive patients on antiretroviral treatment and/or cotrimoxazole prophylaxis use traditional medicine: perceptions of health workers, traditional healers and patients: a study in two provinces of South Africa	Western Cape and KwaZulu-Natal	A number of HIV positive patients on ART concurrently use traditional medicine for various reasons	[74]
Factors associated with patterns of plural healthcare utilization among patients taking antiretroviral therapy in rural and urban South Africa: a cross-sectional study	Not indicated	Increased plural healthcare utilization, inequitably distributed between rural and urban areas, is largely a function of higher socioeconomic status, better ability to finance healthcare and factors related to poor quality of care in ART clinics	[91]
The social and clinical characteristics of patients on antiretroviral therapy who are 'lost to follow-up' in KwaZulu-Natal, South Africa: a prospective study	KwaZulu-Natal	The use of alternative medicine and depression are some of the factors contributing to non-adherence and lost to follow-up	[70]
Traditional complementary and alternative medicine and antiretroviral treatment adherence among HIV patients in KwaZulu-Natal, South Africa	KwaZulu-Natal	The use of herbal treatment reduces ARV adherence. It is therefore recommended that patients' use of TCAM be considered in ARV adherence management	[92]
The cultural and community-level acceptance of antiretroviral therapy among traditional healers in Eastern Cape, South Africa	Eastern Cape	Cultural consistencies between traditional and biomedical medicine may strengthen HIV/AIDS treatment programs to provide ART in resource-poor settings	[92]
“That is why I stopped the ART”: Patients' and providers' perspectives on barriers to and enablers of HIV treatment adherence in a South African workplace programme	Not indicated	HIV positive patients stop the use of ART due to the use of traditional medicines	[93]
Patients consulting traditional health practioners in the context of HIV/AIDS in urban areas in KwaZulu-Natal, South Africa	KwaZulu-Natal	A number of HIV positive patients in heath care at the same time consult traditional health practitioners for various reasons, which affects ART adherence	[73]
Use of traditional complementary and alternative medicine for HIV patients in KwaZulu-Natal, South Africa	KwaZulu-Natal	Traditional herbal therapies and TCAM are commonly used by HIV treatment naive outpatients of public health facilities in South Africa	[71]
Self-reported use of traditional, complementary and over-the-counter medicines by HIV-infected patients on antiretroviral therapy in Pretoria, South Africa	Gauteng	HIV-infected patients on ART in this study used a limited range of over-the-counter products as well as those from traditional, complementary and alternative medicine practices	[94]
Use of traditional medicine by HIV-infected individuals in South Africa in the era of antiretroviral therapy	Not indicated	Traditional medicine use is common among individuals with moderate and advanced HIV disease. Concomitant use with ART has the potential for drug interactions and should be discussed routinely in ART counselling	[69]

Another concern is whether there are interactions between phytochemicals from herbal preparations and ARV and whether or not these negatively impacts the efficacy of ARV, or how do phytochemicals interact indirectly with ARV pharmacokinetics and pharmacodynamics. For example, the powder derived from the leaf of *Moringa oleifera Lam*., has been shown to inhibit cytochrome p450 3A4, 1A2 and 2D6 activity *in-vitro*, but its intake did not alter the pharmacokinetics of nevirapine in a one-sequence cross over study of HIV patients on ART [77]. Members of *Hypoxis hemerocallidea*, known as African potato and commonly used as an alternative in the management of AIDS, potentially inhibit the efflux of indinavir, a protease inhibitor, across human cell lines, and increases indinavir bioavailability in Sprague–Dawley rats [78]. Equally, *Sutherlandia frutescens* has been reported to significantly reduce the availability of another protease inhibitor, atazanavir, in-vitro [79]. The transport of atazanavir is mediated by P-glycoprotein (P-gp), while its metabolism is mediated by CYP3A4 and CYP3A5. Studies have advocated for clinical trials to better understand the herbal-drug interactions with the aim of improving the outcomes of ARV use in South Africa [80, 81]. It is plausible that herb–drug interactions could negatively impact ARV bioavailability and lead to the development of drug resistance and poor viral suppression. Nevertheless, our improved understanding may be hindered by the evolving changes in treatment regimens.

The development of acquired resistance is almost inevitable in antiretroviral therapy and plays a major role in viral suppression [82]. Acquired resistance is also driven by several factors, sometimes intertwined, and efforts aimed at minimizing the development of acquired resistance at the individual and at the population levels are in different fronts. These include, administering the best treatment regimen to provide the highest genetic barrier for viral resistance, maintaining optimal adherence through counselling and programmatic measures, and regular viral load monitoring for timely interventions.

The outcomes described here-in should be appreciated in the background of some limitations. First, psychosocial parameters, such as mental health, stigma and discrimination, which are potential challenges to the desired outcomes of the 90-90-90 strategy were not reviewed for impact. For example, stigma, discrimination and depression may impede willingness for voluntary counselling and testing, and also negatively impact adherence and favourable viral suppression for those on treatment. Second, drug stock-outs and drug toxicities were not reviewed for implications in non-adherence, although allusions on how they impact adherence have been indicated. Third, although we attempted to make this review as complete as possible, the literature search did not include abstracts and conference proceedings, or data presented in theses and dissertations.

## Conclusion

**Fig. 3:**
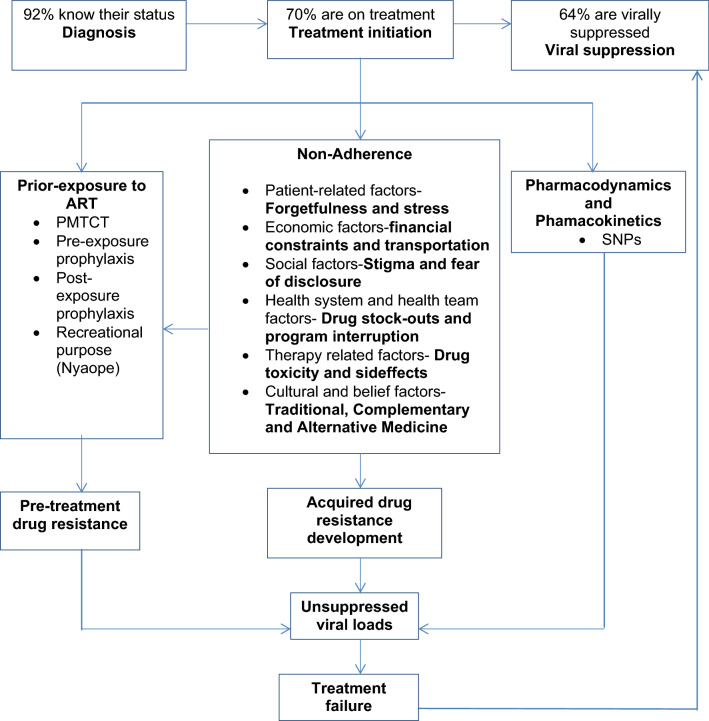
South Africa’s progress towards the 90-90-90 target (http://aidsinfo.unaids.org/, 2019) and factors associated with unsuppressed HIV viral loads

This narrative review provides insights on antiretroviral drug resistance in the pre-treated population in South Africa which has reached moderate levels in specific geographic populations in eight of the nine Provinces. Therefore, in the face of the expanding access to treatment, the implementation of Provincial drug resistance surveillance systems to track trends for intervention is a reasonable proposal to support efforts and sustain the goals of UTT considering local realities. There is copious data showing that TCAM is commonly used concomitantly with ARV resulting in negative impacts on ARV adherence and viral suppression. It is therefore important that TCAM should be taken seriously during counselling sessions for patients on ARV as it directly relates to achieving viral suppression. Also, there should be more objective and coordinated approaches to detect prior exposure to ARV before treatment initiation. In certain populations such as children, pregnant women, males, and those who have been on ART for more than three years, adherence measures should be intensified. On the other hand, there is a dearth of data on host genetics of the very diverse ethnic South African population and its relationship to bioavailability of antiretrovirals. Future investigations should also provide additional data for empirical evidence that the population is accessing the test and treat programme early enough, and that adherence is sustained. Definitely, resistance in the pre-treated population, TCAM, adherence, host genetics are all interwoven on their impact on viral suppression (Fig. 3). Optimal management of these relationships will be beneficial in ensuring sustained viral suppression in at least 90% of those on treatment, a key component of the 90-90-90 strategy.

## Data Availability

All data used for the analysis is available in the manuscript.

## Abbreviations

ARV: Antiretroviral
AZT: Zidovudine
CYP2B: Cytochrome P450 2B6
DRM: Drug resistant mutation
d4t: Stavudine
MDR1: Multi-drug resistance 1
NGS: Next generation sequencing
NRTI: Nucleoside reverse transcriptase inhibitor
NNRTI: Non-nucleoside reverse transcriptase inhibitor
PI: Protease inhibitor
PMTCT: Prevention of mother-to-child transmission
SDRM: Surveilance drug resistance mutation
TAMs: Thymidine analog mutations
TCAM: Traditonal, complementary and alternative medicine
TLD: Tenofovir–Lamuvudine–Dolutegravir
UNAIDS: Joint United Nations Programme on HIV/AIDS
UTT: Universal Test and Treat programme
